# The Interaction Between Iron and Selenium Affects Ferroptosis in Colorectal Cancer

**DOI:** 10.3390/ijms27093963

**Published:** 2026-04-29

**Authors:** Fulin Tao, Menghui He, Yong Dai

**Affiliations:** 1 School of Medicine, Anhui University of Science & Technology, Huainan 232001, China; fulinxerxes@163.com; 2 National Synchrotron Radiation Laboratory, University of Science and Technology of China, Hefei 230026, China; hemenghui@ustc.edu.cn

**Keywords:** colorectal cancer, ferroptosis, iron homeostasis, selenium metabolism, PCBP1/2, NCOA4, ALKBH8, selenoproteins

## Abstract

Colorectal cancer (CRC) remains a major cause of cancer-related death, and resistance to chemotherapy and radiotherapy continues to limit durable disease control. Ferroptosis, an iron-dependent form of cell death driven by lipid peroxidation, has therefore emerged as a potential therapeutic strategy. However, models focused solely on glutathione peroxidase 4 (GPX4) and solute carrier family 7 member 11 (SLC7A11) do not fully explain why CRC cells differ in their sensitivity to ferroptosis. In this review, we examine how ferroptosis in CRC is shaped by iron trafficking and selenium-dependent antioxidant defense. We first discuss the poly(rC)-binding proteins 1 and 2 (PCBP1/2)-nuclear receptor coactivator 4 (NCOA4) axis, which regulates iron storage, trafficking, and ferritinophagy. We then review the AlkB homolog 8 (ALKBH8)-directed selenoprotein network, which supports the detoxification of lipid peroxides and maintenance of redox homeostasis. We next consider how these two systems intersect and how their interplay influences ferroptosis sensitivity. We also discuss why concurrent disruption of iron handling and selenium-dependent defense mechanisms may enhance therapeutic efficacy. Finally, we outline potential clinical applications, including combination strategies and biomarker development.

## 1. Introduction

Ferroptosis is an iron-dependent form of regulated cell death characterized by excessive accumulation of lipid reactive oxygen species (ROS) and uncontrolled lipid peroxidation. Its molecular mechanisms and morphological features differ from those of apoptosis, necroptosis, and autophagy [1,2]. In recent years, ferroptosis has attracted increasing attention, especially in tumors that develop resistance to conventional therapies. This association is particularly relevant in CRC, which is the third most common malignancy worldwide and the second leading cause of cancer-related death. In 2023, there were approximately 2.29 million new cases and 1.11 million deaths worldwide. Moreover, the global burden of CRC has risen markedly over the past three decades, from 916,583 cases in 1990 to more than 2.19 million cases in 2021, and is projected to reach 3.2 million cases by 2040 [3,4,5,6]. Accordingly, ferroptosis has become an especially important area of study because CRC cells display distinctive metabolic features, including elevated intracellular iron content, increased ROS production, and disordered lipid metabolism, all of which may render them particularly vulnerable to ferroptotic death [1,7].

The canonical ferroptosis defense axis is centered on SLC7A11, glutathione (GSH), and GPX4, which together protect cells from lipid peroxidation and ferroptotic injury [8,9]. Within this axis, GPX4, a selenoprotein, reduces phospholipid hydroperoxides, whereas SLC7A11 maintains cystine uptake to support GSH synthesis [8,10]. Although this pathway is crucial, targeting it alone often fails to impose sustained ferroptotic pressure on cancer cells. One major reason is that tumor cells can engage multiple adaptive mechanisms, including reinforcement of the xCT transporter-CD44 variant isoform (CD44v) axis, upregulation of GPX4 and ferroptosis suppressor protein 1 (FSP1), activation of the nuclear factor erythroid 2-related factor 2 (NRF2)-dependent antioxidant program, and mitochondrial remodeling [11,12]. Even when the canonical GPX4/SLC7A11 axis is disrupted, additional pathways, such as the FSP1-coenzyme Q10, dihydroorotate dehydrogenase (DHODH), and GTP cyclohydrolase 1-tetrahydrobiopterin systems, can continue to suppress membrane lipid peroxidation, thereby preserving ferroptosis resistance under GSH-limited conditions [8,11]. Accordingly, previous reviews have argued that reliance on established nodes such as SLC7A11 and NRF2 is insufficient and that additional regulatory layers must be identified in CRC ferroptosis (Table 1) [9,13,14].

At a more fundamental level, ferroptosis can be viewed, in part, through the opposing influences of two essential trace elements, iron and selenium [8,15]. Iron promotes ferroptosis by fueling Fenton-like reactions that generate hydroxyl radicals and initiate uncontrolled lipid autoxidation [8,11]. Importantly, this pro-ferroptotic activity depends on the availability of catalytically active ferrous iron (Fe(II)), which is tightly controlled by intracellular systems for iron storage, trafficking, and release. In contrast, selenium is incorporated into GPX4 and other selenoproteins, which eliminate phospholipid peroxides and thereby inhibit ferroptosis, supporting cell survival [1,8]. Together, these opposing influences provide a useful conceptual framework for considering how trace-element metabolism may modulate ferroptosis sensitivity, while recognizing that additional parallel regulatory systems also contribute to CRC ferroptosis.

Notably, intracellular iron and selenium homeostasis is not maintained by free diffusion. Instead, both elements rely on dedicated chaperones or transport systems to ensure safe trafficking, limit trace-element toxicity, and preserve the function of downstream protein networks. In the iron pathway, ferritinophagy, a selective autophagic process mediated by NCOA4, releases ferritin-stored iron into the labile iron pool (LIP) and is therefore a major determinant of intracellular iron flux [16,17]. More broadly, iron homeostasis in CRC also involves transferrin-dependent iron uptake, DMT1-mediated intracellular transport, ferroportin-dependent iron export, ferritin storage dynamics, and hepcidin-associated systemic regulation, all of which can influence the labile iron pool and thereby modify ferroptosis sensitivity. Within this framework, PCBP1/2 act as cytoplasmic iron chaperones that bind iron and deliver it to target proteins. Importantly, PCBP1 can interact with NCOA4 to facilitate iron loading into ferritin, suggesting that the PCBP1/NCOA4-ferritin axis may contribute to, rather than fully define, sensitivity to iron-driven cell death [18,19]. On the selenium side, emerging evidence suggests that peroxiredoxin 6 (PRDX6) may serve as a candidate modifier linking redox adaptation, phospholipid metabolism, and selenium-related ferroptosis regulation, although its direct role in selenium handling or selenoprotein biosynthetic control remains hypothetical and requires further validation in CRC [20,21,22,23]. At the same time, ALKBH8, a transfer RNA methyltransferase, acts as an important upstream regulator of selenocysteine recoding efficiency by modifying specific transfer RNAs required for selenocysteine incorporation. Because ALKBH8 depletion impairs the expression or activity of selected ferroptosis-relevant selenoproteins and induces ferroptosis in CRC cells, this regulatory layer may represent a therapeutically relevant vulnerability in CRC [16].

Importantly, the present review is intended as a focused conceptual discussion rather than an exhaustive map of all ferroptosis-regulatory systems in CRC. Beyond the canonical GPX4–glutathione axis, ferroptosis is also shaped by multiple parallel regulatory mechanisms. These include GPX4-independent defense pathways such as FSP1-CoQ10 and DHODH [24,25,26], lipid-remodeling processes involving ACSL4 and LPCAT3 [27,28], and NADPH-linked redox metabolism [29]. Together, these pathways can modify ferroptosis sensitivity in parallel with the iron- and selenium-related processes emphasized in this review, rather than being fully captured by a single regulatory model [30,31]. We therefore use the iron–selenium framework as a selective interpretive lens to examine how trace-element-related processes may modify ferroptosis sensitivity in CRC within this broader regulatory landscape, rather than as a complete or sufficient model of ferroptosis regulation.

Against this background, this review focuses on trace-element chaperone systems that regulate iron and selenium metabolism beyond the canonical GPX4/SLC7A11 pathway in CRC. We first examine the context-dependent role of PCBP1/2 in ferroptosis, considering evidence that these proteins may either buffer free iron and suppress ferroptosis or, under specific stress conditions, facilitate ferritin-associated iron mobilization and thereby intensify ferroptotic stress [19,32,33]. We then discuss the ALKBH8-centered selenoprotein network, including GPX4-independent defense mechanisms mediated by selenoproteins such as selenoprotein I (SELENOI) and thioredoxin reductase 1 (TXNRD1) [16,20].

**Figure 1:**
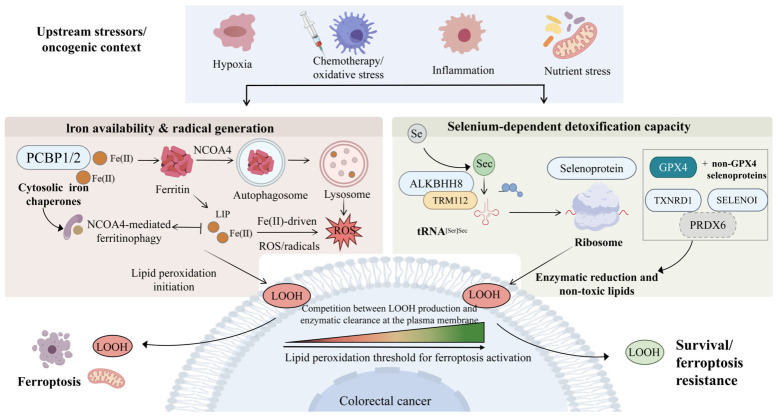
Conceptual iron- and selenium-centered framework for ferroptosis sensitivity in colorectal cancer. Upstream stressors in the oncogenic context, including hypoxia, chemotherapy/oxidative stress, inflammation, and nutrient stress, may reshape the balance between iron availability and selenium-dependent detoxification capacity. On the iron side, PCBP1/2-mediated cytosolic iron chaperoning and NCOA4-dependent ferritinophagy are shown as representative processes that can influence ferritin iron mobilization, expand the labile iron pool, and promote Fe(II)-associated reactive oxygen species generation. On the selenium side, selenium utilization through the ALKBH8–TRM112–tRNA^[Ser]Sec^ machinery is depicted as supporting the synthesis of GPX4 and selected selenoproteins, including TXNRD1, SELENOI, and the candidate modifier PRDX6, thereby contributing to enzymatic detoxification of lipid hydroperoxides. These pathways are shown as functionally convergent at the level of lipid peroxide generation and detoxification capacity, rather than as a fully established linear signaling module. The balance between LOOH production and enzymatic clearance may influence whether colorectal cancer cells undergo ferroptosis or maintain survival and ferroptosis resistance. Abbreviations: PCBP1/2, poly(rC)-binding proteins 1 and 2; NCOA4, nuclear receptor coactivator 4; Fe(II), ferrous iron; LIP, labile iron pool; ROS, reactive oxygen species; Se, selenium; Sec, selenocysteine; ALKBH8, AlkB homolog 8; TRM112, TRM112 methyltransferase activator homolog; tRNA^[Ser]Sec^, selenocysteine-specific transfer RNA initially aminoacylated with serine; GPX4, glutathione peroxidase 4; TXNRD1, thioredoxin reductase 1; SELENOI, selenoprotein I; PRDX6, peroxiredoxin 6; LOOH, lipid hydroperoxide(s).

Although the regulatory mechanisms governed by PCBP1/2 and ALKBH8 are not exclusive to colorectal cancer and have been documented in other malignancies, they may be of particular biological relevance in the colorectal setting. In CRC, ferroptosis is modulated by multiple tumor microenvironmental and context-specific factors. Chronic luminal iron exposure can epigenetically alter the expression of NRF2 target genes in colonic epithelial cells, disrupt intracellular iron homeostasis, and thereby change the susceptibility to ferroptosis [34,35,36]. The gut microbiota also participates in this regulatory network; for instance, trans-3-indoleacrylic acid produced by Peptostreptococcus anaerobius strengthens ferroptosis resistance via the aryl hydrocarbon receptor (AHR)–aldehyde dehydrogenase 1 family member A3 (ALDH1A3)–ferroptosis suppressor protein 1 (FSP1)/coenzyme Q10 (CoQ10) signaling axis [37,38]. Meanwhile, hypoxia induces a ferroptosis-sensitive phenotype by upregulating iron metabolism-related genes such as divalent metal transporter 1 through hypoxia-inducible factor 2α [39,40]. Common oncogenic driver mutations further intersect with ferroptosis pathways, notably APC inactivation leading to constitutive Wnt/β-catenin activation, as well as KRAS and TP53 alterations that modulate FSP1, SLC7A11, and lipid metabolism. Together, these factors establish a CRC-specific regulatory landscape for the iron–selenium network, supporting the need to incorporate intestinal ecology and molecular subtypes into investigations of the PCBP1/2-NCOA4 and ALKBH8 axes. Finally, we outline potential translational implications, including dual-target strategies such as acevaltrate (ACE), the emerging use of ferroptosis-related gene (FRG) signatures for prognosis and treatment stratification, and broader exploratory efforts aimed at overcoming therapy resistance by modulating trace-element metabolism (Figure 1) [41,42,43,44]. This perspective should be interpreted in the context of CRC heterogeneity, tumor microenvironmental influences, lipid-remodeling programs, and the still limited in vivo and clinical evidence supporting ferroptosis-directed therapeutic translation.

## 2. The PCBP1/2-NCOA4 Axis

PCBP1/2 were originally identified as ribonucleic acid/deoxyribonucleic acid (RNA/DNA)-binding proteins, but they are now recognized as key cytosolic iron chaperones in mammalian cells [45,46]. Both PCBP1 and PCBP2 bind iron and form homo- or heterodimeric complexes, and each contains amino acid residues required for iron coordination, thereby providing a structural basis for iron-chaperone activity [45,46]. Functionally, PCBP1 binds iron–GSH complexes and delivers iron to multiple downstream targets, including ferritin and iron-dependent enzymes [47,48]. Importantly, PCBP1 and PCBP2 are not fully interchangeable in vivo. Instead, they perform distinct, nonredundant functions: PCBP1 is more closely associated with ferritin-directed iron delivery and storage, whereas PCBP2 participates more broadly in divalent metal transporter 1-mediated iron import, ferroportin 1-mediated iron export, and heme oxygenase-1-related heme degradation, together shaping the intracellular availability of catalytic iron [30]. Furthermore, PCBP function is itself influenced by iron status. For example, iron chelation enhances the binding of PCBP1 to RNA, indicating that iron sensing and post-transcriptional regulation are mechanistically linked [49].

Overall, the effects of PCBP1/2 on ferroptosis appear to be context-dependent. Available evidence supports a protective role in iron buffering under some conditions, whereas pro-ferroptotic effects may emerge in specific stress settings, potentially in association with ferritinophagy-related processes. Even under physiological conditions without overt iron overload, Fe(II) that is not effectively sequestered by partner proteins can participate in Fenton chemistry, thereby exacerbating lipid peroxidation and increasing susceptibility to ferroptosis [35]. PCBP-mediated iron buffering therefore helps counter iron toxicity. Consistent with this view, deletion of PCBP1 in mouse liver increases free iron levels, enhances ROS generation, heightens sensitivity to iron and pro-oxidants, and leads to accumulation of oxidized lipids [50]. PCBP1 has been reported to bind iron–GSH complexes in selected models, but the mechanistic relevance of this interaction to ferroptosis regulation remains incompletely defined. For example, plasma-activated Ringer’s lactate alters PCBP1/2 function, disrupts cytoplasmic Fe(II) homeostasis, and thereby impairs GSH-dependent control of iron balance [19]. Similarly, PCBP1 overexpression alleviates iron overload and reduces ferroptotic phenotypes by preserving endothelial iron homeostasis [51]. A comparable protective role has also been observed in astrocytes, where PCBP1 overexpression, like ferroptosis inhibition itself, reverses stress-induced cell death by limiting iron-dependent lipid peroxidation [52].

Notably, the role of PCBP1 is not exclusively anti-ferroptotic. Under specific conditions, it can also promote ferroptosis by participating in NCOA4-mediated ferritinophagy. As a selective autophagy receptor, NCOA4 binds ferritin and promotes its lysosomal degradation, thereby releasing stored iron and increasing the LIP [53,54]. During this process, PCBP1 and NCOA4 cooperatively regulate iron trafficking through ferritin, and loss of either protein reduces this trafficking efficiency, indicating functional synergy in iron metabolism [55]. During ferroptosis induction, PCBP1/NCOA4-dependent ferritinophagy appears to be activated at later stages, further increasing cytoplasmic catalytic Fe(II) and thereby intensifying ferroptotic pressure [19,56]. By contrast, under oxidative stress conditions, PCBP2 promotes iron transport to mitochondria and induces mitochondrial dysfunction, suggesting that the ferroptosis-promoting activities of PCBP1 and PCBP2 are related but not identical [19,57]. However, direct evidence supporting a unified biphasic PCBP1/2-NCOA4 switch remains limited, and the specific conditions under which PCBP1/2 shift from protective to pro-ferroptotic roles remain to be clarified.

Overall, the dual effects of PCBP1/2 on ferroptosis reflect both temporal dynamics and context dependence. In the early phase of oxidative stress, PCBP1/2 bind and safely traffic Fe(II), reducing ROS generation and protecting cells from iron toxicity [33,50]. In later phases, however, interaction between PCBP1 and NCOA4 can favor ferritinophagy, increase the LIP, and ultimately drive ferroptosis [19]. This biphasic behavior has important therapeutic implications because it suggests that the consequences of targeting PCBP1/2 may vary with stress state and disease context. Supporting this view, PCBP1 knockdown promotes polyunsaturated fatty acid peroxidation by upregulating ALOX15 and concurrently causes iron accumulation and mitochondrial dysfunction [41]. In head and neck cancer, PCBP1 also suppresses ferritinophagy-related ferroptosis by binding the 3′-untranslated region of BECN1 messenger RNA, thereby inhibiting autophagy activation and reducing ALOX15 expression [58]. Consistently, the ferroptosis inducer sulfasalazine shows stronger tumor-suppressive effects in xenografts derived from PCBP1-silenced cancer cells, suggesting that loss of PCBP1 can sensitize tumors to ferroptosis induction (Table 2) [58].

**Figure 2:**
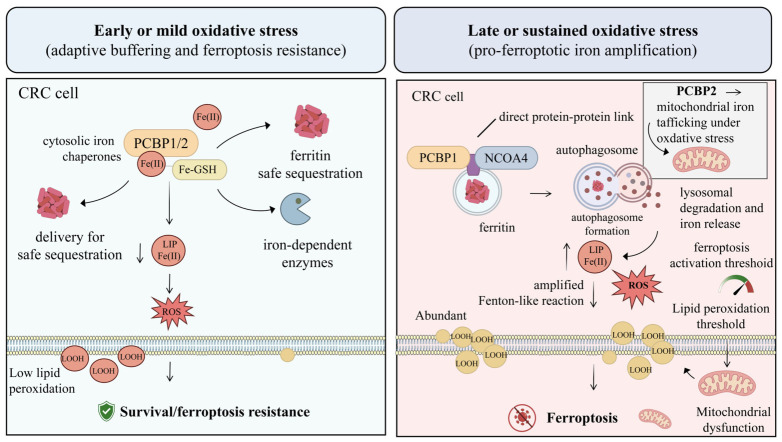
Biphasic role of the PCBP1/2–NCOA4 axis during oxidative stress in colorectal cancer cells. Under early or mild oxidative stress, PCBP1/2 acts predominantly as a cytosolic iron chaperone system that binds Fe(II), cooperates with glutathione-associated iron buffering, and delivers iron to ferritin and other iron-dependent proteins for safe sequestration or controlled utilization. This buffering function limits expansion of the labile iron pool, restrains reactive oxygen species generation, and maintains low lipid peroxidation, thereby promoting survival and ferroptosis resistance. Under late or sustained oxidative stress, however, PCBP1 cooperates with NCOA4 to facilitate ferritinophagy, increasing ferritin degradation, lysosomal iron release, and the labile iron pool, which amplifies Fenton-like reactions, reactive oxygen species accumulation, and membrane lipid hydroperoxide formation. In parallel, PCBP2-mediated mitochondrial iron trafficking may further contribute to mitochondrial dysfunction. Together, these changes lower the effective threshold for ferroptotic damage and shift colorectal cancer cells from adaptive buffering toward ferroptosis. Abbreviations: CRC, colorectal cancer; PCBP1/2, poly(rC)-binding proteins 1 and 2; Fe(II), ferrous iron; Fe–GSH, ferrous iron–glutathione complex; LIP, labile iron pool; ROS, reactive oxygen species; NCOA4, nuclear receptor coactivator 4; LOOH, lipid hydroperoxide(s).

Whether PCBP1/2 can serve as therapeutic targets for ferroptosis modulation in CRC remains to be systematically tested in CRC-specific models [1]. Nevertheless, current evidence already points to their relevance in this disease. For example, the ZDHHC9/PCBP1/SLC7A11 signaling axis has been linked to ferroptosis regulation and tumor metastasis in CRC [59,60]. In addition, FRG signatures containing iron-metabolism regulators have been identified in CRC, and expression of genes such as ataxia-telangiectasia mutated kinase (ATM), a key regulator of NCOA4-mediated ferritinophagy, correlates with both immune-cell infiltration and patient prognosis [42]. Together, these findings position PCBP1/2 as important nodes in the CRC ferroptosis network and support their potential value as targets for overcoming treatment resistance (Figure 2). Although these observations suggest potential relevance to ferroptosis-targeted therapy, CRC-specific experimental validation remains limited, and therapeutic conclusions should therefore be interpreted cautiously.

## 3. Ferritinophagy and the NCOA4-FTH1 Axis

NCOA4 is a selective autophagic receptor that mediates ferritinophagy and represents an important component of cellular iron homeostasis [61,62]. Ferritin is a nanocage composed of 24 subunits, ferritin heavy chain 1 (FTH1) and ferritin light chain (FTL), that stores intracellular iron. When iron demand rises or cellular iron becomes limiting, ferritin can be delivered to lysosomes for degradation and iron release through either macroautophagy or endosomal microautophagy mediated by the endosomal sorting complexes required for transport machinery [61]. Through this process, the NCOA4-FTH1 axis mobilizes ferritin-bound iron, increases the LIP and catalytic Fe(II), and thereby enhances lipid peroxide generation and ferroptosis. Although ferritinophagy is an important determinant of intracellular iron mobilization, the labile iron pool is also influenced by iron uptake, export, storage, and systemic regulatory signals, including transferrin-dependent transport, DMT1, ferroportin, and hepcidin-related control.

Structural studies have clarified how this system operates at the molecular level. Cryo-electron microscopy has resolved the binding interface between NCOA4 and FTH1 and identified key NCOA4 residues required for interaction with the hydrophobic surface of ferritin [62]. Thermodynamic analyses further indicate that NCOA4 preferentially binds H-rich ferritin assemblies [63]. Recent studies suggest that phase separation may serve as one context-dependent organizational mechanism facilitating ferritin handling, rather than a universal requirement for ferritinophagy in all settings. Homodimeric NCOA4 and multivalent interactions between ferritin particles and NCOA4 may promote the formation of condensates that can be engulfed by autophagosomes or endosomes, thereby facilitating ferritinophagy in selected settings [61].

NCOA4 abundance is also tightly regulated, and this regulatory layer is crucial for determining how much iron is released from ferritin. Under iron-replete conditions, HECT and RLD domain containing E3 ubiquitin protein ligase 2 (HERC2) targets NCOA4 for proteasomal degradation [64,65]. Studies have shown that the HERC2-binding region of NCOA4 contains a [2Fe-2S] cluster and can exist in both cluster-bound and cluster-free conformations [64]. HERC2 preferentially recognizes the cluster-bound form of NCOA4 through coordinated interactions involving its Cullin-7-PARC-HERC2 domain and an iron–sulfur cluster-dependent NCOA4-binding domain [64]. These findings explain how NCOA4 senses intracellular iron and couples that information to its own turnover. This regulatory circuit is also oxygen sensitive: under hypoxic conditions, iron–sulfur cluster-mediated NCOA4 degradation is enhanced, whereas at higher oxygen levels NCOA4 is more prone to aggregate and initiate ferritin degradation, indicating that the NCOA4-ferritin axis responds to both iron and oxygen [66]. In addition, the ferritin iron core itself can enhance NCOA4 binding to ferritin [67]. Taken together, HERC2-mediated NCOA4 degradation is a key checkpoint in iron mobilization. When NCOA4 stability increases or its interaction with ferritin is strengthened, lipid peroxidation intensifies and ferroptosis is promoted.

NCOA4 is also modulated by other signaling pathways. ATM-dependent regulation of NCOA4 has been proposed in specific models and may provide one mechanistic link between DNA damage signaling and ferritinophagy, although the generality of this axis across contexts remains to be fully established [56]. As a core sensor of DNA double-strand breaks, ATM promotes ferritinophagy and ferroptosis, at least in part, by phosphorylating NCOA4, strengthening the interaction between NCOA4 and ferritin, and enhancing intracellular iron recycling [56]. Accordingly, the ATM–NCOA4 relationship may represent one model-dependent link between genome surveillance and ferroptosis susceptibility, and ATM-deficiency-associated resistance to ferroptosis may be, at least in part, independent of p53 [56]. At the same time, NCOA4 behavior is highly context-dependent. Under iron-replete conditions, iron binding to an intrinsically disordered region of NCOA4 can drive formation of insoluble aggregates, causing NCOA4 to dissociate from ferritin and promoting early ferritin accumulation during iron excess [68]. During prolonged iron supplementation, however, NCOA4 condensates can deliver ferritin to lysosomes through a noncanonical TAX1BP1-dependent pathway, thereby preventing excessive iron accumulation and subsequent functional iron deficiency [68]. NRF2 also participates in this network through HERC2 and VAMP8; in cells lacking NFE2L2/NRF2, reduced HERC2 expression leads to concurrent accumulation of ferritin and NCOA4 and increases ferroptosis sensitivity [69]. Overall, these findings indicate that NCOA4 is not merely a degradative factor. Rather, its effects on iron homeostasis and ferroptosis depend on the combined influences of iron status, oxygen availability, and DNA damage context.

In CRC, the ferritinophagy–ferroptosis axis has emerged as a particularly attractive therapeutic target, and several experimental inducers have already been described. Sodium butyrate (NaB), a metabolite derived from dietary fiber, induces ferroptosis in CRC HCT-116 and Caco-2 cells by promoting NCOA4-FTH1-mediated ferritinophagy, leading to reduced proliferation, increased Fe(II), elevated lipid ROS, and abnormal mitochondrial morphology, while showing little toxicity in normal FHC cells [8]. Mechanistically, NaB downregulates FTH1, increases lysosomal Fe(II), and enhances colocalization of NCOA4 with FTH1; in xenograft models, it also suppresses tumor growth while increasing intratumoral Fe(II) and NCOA4 expression [8]. Additional evidence supports this concept. Cytoglobin enhances ferroptosis by promoting ferritin degradation in CRC cells, and cytoglobin-overexpressing cells show increased lysosomal signaling together with stronger colocalization of NCOA4 and LC3B [70]. NCOA4-mediated ferroptosis also appears to inhibit epithelial–mesenchymal transition through the phosphatidylinositol 3-kinase/protein kinase B/mechanistic target of rapamycin pathway, as NCOA4 downregulation promotes mesenchymal marker expression in CRC cells [71]. Moreover, CRC-directed ferritinophagy inducers have already been developed, including the sinomenine-derived compound D3-3, which strengthens the FTH1-NCOA4 interaction and effectively triggers ferroptosis while suppressing tumor growth in HCT-116 xenografts [54].

**Figure 3:**
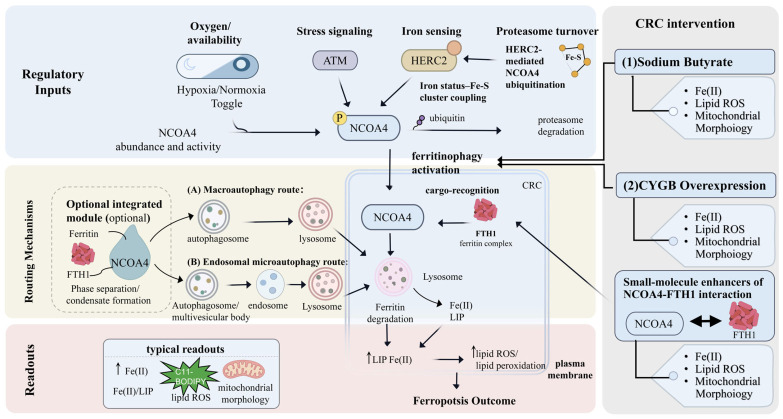
Regulatory, mechanistic, and translational landscape of the NCOA4–FTH1 ferritinophagy axis in colorectal cancer. NCOA4-mediated ferritinophagy in colorectal cancer (CRC) is controlled by multiple upstream regulatory inputs, including oxygen availability, model-dependent stress signaling through ATM, and iron sensing linked to HERC2-mediated ubiquitin–proteasome turnover. Once activated, NCOA4 recognizes the ferritin complex through FTH1 and directs ferritin toward lysosomal degradation through either macroautophagy or endosomal microautophagy, with phase separation/condensate formation serving as an optional organizational module. Ferritin degradation increases the labile ferrous iron pool, promotes lipid reactive oxygen species accumulation and lipid peroxidation at the plasma membrane, and thereby drives ferroptotic outcome. Typical experimental readouts include increased Fe(II)/LIP, elevated lipid ROS, and abnormal mitochondrial morphology. In CRC models, sodium butyrate, CYGB overexpression, and small-molecule enhancers of NCOA4–FTH1 interaction represent candidate interventions that converge on this axis to promote ferritinophagy-dependent ferroptosis. Abbreviations: CRC, colorectal cancer; ATM, ataxia-telangiectasia mutated; HERC2, HECT and RLD domain containing E3 ubiquitin protein ligase 2; Fe-S, iron–sulfur; NCOA4, nuclear receptor coactivator 4; FTH1, ferritin heavy chain 1; Fe(II), ferrous iron; LIP, labile iron pool; ROS, reactive oxygen species; CYGB, cytoglobin; ↑, upregulation.

From a translational perspective, the ferritinophagy–ferroptosis axis offers multiple intervention points for CRC treatment. Autophagy promotes ferroptosis in part by degrading ferritin, and deletion of ATG5 or ATG7 reduces intracellular Fe(II) and lipid peroxidation, thereby significantly limiting erastin-induced ferroptosis, whereas NCOA4 overexpression has the opposite effect [72,73]. Autophagosome-tethering compounds (ATTECs) provide an especially innovative strategy. For example, 16AA-C10-DPC combines an NCOA4-derived ferritin-binding peptide with an LC3-binding domain, enabling selective ferritin degradation through the autophagy-lysosome pathway, release of endogenous Fe(II), and potent ferroptosis induction in CRC cells [74]. At the same time, ferritinophagy should be viewed as a double-edged sword: under physiological conditions it helps maintain iron homeostasis, whereas under pathological conditions excessive activation releases large amounts of free iron, drives lipid peroxidation, and culminates in ferroptotic cell death [54,75]. For this reason, translational strategies centered on the NCOA4-FTH1 axis should prioritize careful definition of the therapeutic window and development of pharmacodynamic monitoring systems. Combining this axis with other ferroptosis-inducing nodes may further help overcome resistance associated with single-target approaches. Importantly, the NCOA4-FTH1 binding interface has now been structurally defined, providing a rational molecular basis for the design of ferritinophagy modulators. However, the translational relevance of this axis should be interpreted cautiously, given current limitations in CRC-specific validation, systemic toxicity risk, metabolic compensation, and potential heterogeneity in tumor and normal-tissue responses to global iron-homeostasis modulation (Figure 3) [62].

## 4. The Selenium Chaperone System and the Selenoproteome

Selenocysteine, the twenty-first genetically encoded amino acid, is a core component of selenoproteins and is essential for many redox reactions [76,77]. Its incorporation during translation requires recoding of the UGA stop codon, a process coordinated by the selenocysteine insertion sequence element in the 3′-untranslated region, SECIS-binding protein 2, the elongation factor EEFSEC, and transfer RNA^[Ser]Sec^ [76,77,78]. The efficiency of this recoding step is strongly influenced by modifications within the anticodon loop of transfer RNA^[Ser]Sec^, which vary across tissues and according to selenium availability. Within this biosynthetic framework, ALKBH8 acts as an important upstream regulator. After forming a complex with the cofactor TRM112, ALKBH8 catalyzes formation of 5-methoxycarbonylmethyluridine and thereby provides the basis for subsequent formation of 5-methoxycarbonylmethyl-2-thiouridine or 5-methoxycarbonylmethyluridine-2′-O-methylribose [79,80,81]. ALKBH8 is best viewed as an important upstream regulator of selenocysteine recoding efficiency and of selected selenoprotein outputs relevant to ferroptosis, rather than as a uniform master regulator of the entire selenoproteome.

Although ALKBH8 knockout does not always cause an obvious global phenotype, it disrupts transfer RNA^[Ser]Sec^ modification and significantly reduces UGA recoding efficiency in specific selenoproteins [80]. Under oxidative stress conditions, ROS induces ALKBH8 expression and thereby promotes selenoprotein synthesis [82]. In normal cells, stress increases transfer RNA modifications such as 5-methoxycarbonylmethyluridine-2′-O-methylribose, thereby helping maintain antioxidant capacity. By contrast, ALKBH8 deficiency disrupts this transfer RNA reprogramming and impairs selenoprotein synthesis [82]. In CRC cells, loss of ALKBH8 broadly reduces selenoprotein expression and activity and induces ferroptosis, suggesting that the selenoproteome may constitute a therapeutic vulnerability [16]. Beyond ferroptosis, ALKBH8 deficiency also promotes cellular senescence, mitochondrial reprogramming, and a more glycolytic metabolic state [83,84].

Although GPX4 is a well-established anti-ferroptotic selenoprotein, recent studies indicate that ferroptosis sensitivity is determined by a broader selenium-dependent network [85]. For example, SELENOI regulates ferroptosis independently of GPX4 in both colitis and CRC [86]. Intestinal epithelial cell-specific deletion of SELENOI induces ferroptosis and suppresses colon tumor growth. Notably, GPX4 overexpression cannot rescue ferroptosis caused by SELENOI loss, whereas SELENOI overexpression can partially counteract ferroptosis induced by GPX4 deletion [86]. TXNRD1 represents another major selenoprotein node, is highly expressed in many tumors, and helps maintain redox homeostasis [87]. Although broader redox interactions have been proposed for RSL3 and ML162, GPX4 remains their best-established ferroptosis-relevant target, and any contribution of TXNRD1 should therefore be interpreted cautiously [88]. Taken together, GPX4, TXNRD1, and PRDX6 should be regarded as functionally distinct and only partially intersecting components of selenium-linked redox defense, rather than as a uniform equivalent anti-ferroptotic network [85]. PRDX6 may represent a candidate link between redox adaptation, phospholipid metabolism, and selenium-related ferroptosis regulation; however, its direct role in selenium handling or selenoprotein biosynthetic control remains hypothetical and requires further validation, particularly in CRC. Studies have suggested a functional association between PRDX6 and intracellular selenium utilization or selenoprotein-related processes, although the directionality and mechanistic basis of this relationship remain incompletely defined. PRDX6 depletion lowers selenoprotein abundance and increases ferroptosis sensitivity. However, questions of whether PRDX6 serves as a broadly acting intracellular selenium transporter, and how it functions in CRC specifically, remain unresolved questions that require further study [17,18,89]. Co-expression network analyses further support a functional association between PRDX6 and the machinery for selenocysteine synthesis (Table 3) [90]. These selenium-linked mechanisms should be interpreted within a broader ferroptosis-regulatory network that also includes selenium-independent systems such as FSP1-CoQ10, lipid-remodeling pathways, iron metabolism, and NADPH-dependent redox processes.

**Figure 4:**
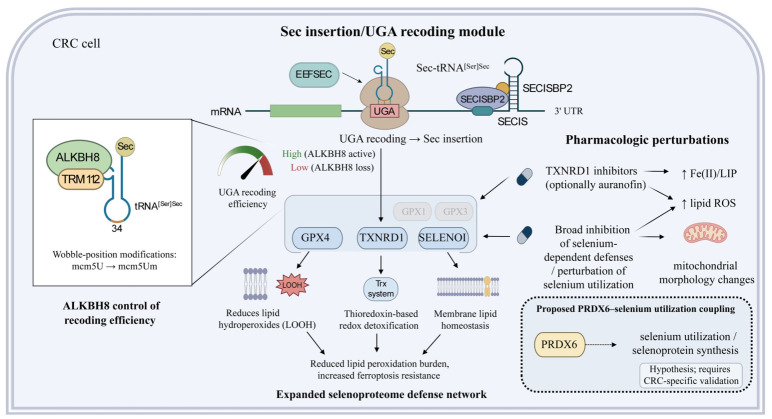
ALKBH8-centered control of selenoprotein biosynthesis and selenium-dependent ferroptosis defense in colorectal cancer. In colorectal cancer (CRC) cells, selenocysteine (Sec) incorporation depends on UGA recoding machinery composed of the SECIS element, SECISBP2, EEFSEC, and tRNA^[Ser]Sec^. ALKBH8, together with its cofactor TRM112, modifies tRNA^[Ser]Sec^ and thereby regulates the efficiency of UGA recoding and Sec insertion. When ALKBH8 activity is preserved, the expression of a broader selenoprotein defense network—including GPX4, TXNRD1, and SELENOI—is maintained, supporting lipid hydroperoxide detoxification, thioredoxin-dependent redox control, membrane lipid homeostasis, and ferroptosis resistance. Loss of ALKBH8 reduces recoding efficiency and weakens this selenium-dependent protective network. The figure also summarizes pharmacologic vulnerabilities, including TXNRD1 inhibition and broader disruption of selenium utilization, which may increase Fe(II)/LIP, elevate lipid ROS, alter mitochondrial morphology, and sensitize CRC cells to ferroptosis. PRDX6 is shown as a candidate modifier associated with redox adaptation and phospholipid metabolism, although its direct role in selenium utilization and CRC ferroptosis remains to be fully validated. Abbreviations: CRC, colorectal cancer; Sec, selenocysteine; UGA, UGA stop codon recoded for selenocysteine insertion; EEFSEC, eukaryotic elongation factor for selenocysteine; tRNA^[Ser]Sec^, selenocysteine-specific transfer RNA initially aminoacylated with serine; SECIS, selenocysteine insertion sequence; SECISBP2, SECIS-binding protein 2; ALKBH8, AlkB homolog 8; TRM112, TRM112 methyltransferase activator homolog; GPX4, glutathione peroxidase 4; TXNRD1, thioredoxin reductase 1; SELENOI, selenoprotein I; PRDX6, peroxiredoxin 6; Trx, thioredoxin; Fe(II), ferrous iron; LIP, labile iron pool; ROS, reactive oxygen species; LOOH, lipid hydroperoxide(s); UTR, untranslated region; ↑, upregulation.

Because tumor cells may rely on selenium metabolism and selected selenoprotein functions, interventions targeting this system have attracted growing research interest [21]. For example, selenoprotein P (SELENOP) facilitates selenium transport and increases resistance to ferroptosis in certain tumors [21]. Simultaneous modulation of multiple selenoprotein-related pathways may prove more effective than single-target intervention in selected contexts, although such strategies require careful evaluation of systemic toxicity and compensatory antioxidant responses [85,91]. Targeting TXNRD1 has attracted particular interest. Compared with broad-spectrum thioredoxin reductase inhibitors, selective inhibition of TXNRD1 may offer greater specificity [92,93]. Overall, ALKBH8-mediated epitranscriptomic regulation provides a useful framework for considering how selenoprotein biology may influence ferroptosis in CRC, whereas PRDX6 is more cautiously discussed as a candidate modifier linked to redox adaptation and phospholipid metabolism rather than as an established regulator of selenium handling (Figure 4).

## 5. Interaction Between Iron and Selenium Metabolic Pathways

Rather than constituting a directly unified signaling circuit, iron-related and selenium-related pathways are more appropriately viewed as distinct regulatory layers that can converge functionally on ferroptosis execution [15,94]. Their most evident point of intersection lies at the level of membrane lipid peroxidation, where iron availability promotes oxidative damage and antioxidant systems determine detoxification capacity. In general, ferroptosis is triggered when three conditions are met simultaneously: continuous generation of lipid hydroperoxides (LOOH) from polyunsaturated phospholipids, impaired enzymatic detoxification of LOOH, and sufficient availability of Fe(II) within the LIP [95]. This is why membrane lipid peroxidation represents the key point of intersection between iron and selenium biology. Iron-related pathways lower the ferroptosis threshold by increasing Fe(II) availability and free-radical generation, thereby accelerating lipid peroxidation. In contrast, selenium-related pathways act in the opposite direction: through GPX4 and other selenoproteins, they support enzymatic reduction and clearance of LOOH, raise the ferroptosis threshold, and enhance cellular resistance to lipid peroxidation [15,17,96].

From this perspective, the PCBP1/2–NCOA4 and ALKBH8-centered pathways are more appropriately viewed as biologically distinct but functionally intersecting regulatory layers that may influence ferroptosis sensitivity in CRC [16,50,55]. PCBP1/2 and NCOA4 primarily promote initiation of Fe(II)-dependent ferroptosis by controlling LIP availability and iron release [55]. By contrast, the ALKBH8-centered selenoprotein network contributes to antioxidant capacity by preserving the expression and function of selected ferroptosis-relevant selenoproteins, including GPX4-mediated detoxification of LOOH, whereas PRDX6 is discussed here as a candidate modifier whose reported association with selenium utilization remains indirect [18,79]. Although these systems operate through distinct molecular mechanisms, they ultimately converge on the regulation of membrane lipid peroxidation. Iron released through ferritinophagy or escaping PCBP-mediated buffering fuels Fenton chemistry, whereas lipid peroxide detoxification is supported primarily by GPX4 and other selenoprotein-linked antioxidant systems within the broader ALKBH8-centered network [16,50].

PRDX6 is discussed here as a candidate modifier rather than as a definitive bridging node, because its reported association with selenium utilization and selenoprotein regulation remains indirect and context-dependent. PRDX6 can limit iron toxicity and suppress ferroptosis by improving intracellular selenium utilization, whereas its depletion decreases selenoprotein expression and induces ferroptosis through GPX4 downregulation [18]. Mechanistically, PRDX6 has been proposed to influence selenoprotein expression through functional connections with selenium utilization and transfer RNA^[Ser]Sec^-related processes [18]. At the same time, PRDX6 is a multifunctional moonlighting enzyme with glutathione peroxidase activity, acidic calcium-independent phospholipase A2 activity, and lysophosphatidylcholine acyltransferase activity, each of which may affect ferroptosis sensitivity through distinct routes [17]. Co-essentiality analyses further indicate that proteins involved in selenocysteine biosynthesis are functionally associated with PRDX6, supporting the concept that the selenocysteine machinery not only sustains GPX4 and TXNRD1 but also connects functionally to PRDX6 [90]. In this context, GPX4, TXNRD1, and PRDX6 are better regarded as functionally distinct and only partially intersecting components of selenium-linked redox defense, rather than as a uniform equivalent anti-ferroptotic axis [85].

NRF2 should be regarded as a context-dependent regulator intersecting with multiple antioxidant, iron-metabolic, and lipid-metabolic programs, rather than as a universal linear integrator of iron–selenium metabolism. Through its antagonistic interplay with BACH1, NRF2 can act as either an anti-ferroptotic regulator or, in certain settings, a pro-ferroptotic regulator by controlling genes involved in labile iron handling, the GSH-GPX4 pathway, and the FSP1-coenzyme Q system [97]. For example, NRF2 helps maintain iron homeostasis through HERC2 and VAMP8, and NRF2 loss disrupts iron metabolism and increases ferroptosis sensitivity [69]. Conversely, excessive dietary iron and systemic iron overload can inhibit NRF2 binding to antioxidant response elements in the GPX4 and SLC7A11 promoters, thereby promoting ferroptosis through transcriptional repression [98]. More broadly, NRF2 suppresses ferroptosis through coordinated regulation of antioxidant, iron-metabolic, and lipid-metabolic programs [99]. In hepatocellular carcinoma, NRF2 has also been shown to reshape selenium metabolism by suppressing SELENOP, promoting intracellular selenium retention, and selectively upregulating GPX4 and TXNRD1. This mechanism enhances resistance to ferroptosis and may suggest additional therapeutic strategies relevant to CRC [100]. At the same time, ferroptosis sensitivity in CRC is also shaped by multiple parallel regulatory mechanisms beyond the iron–selenium axis alone. These include GPX4-independent defense pathways such as FSP1–CoQ10 and DHODH, lipid-remodeling pathways involving ACSL4/LPCAT3, and NADPH-linked redox metabolism [101]. Rather than being fully captured by a single regulatory model, these systems may operate independently, cooperatively, or compensatorily with the iron- and selenium-related processes emphasized in this review [102].

In addition to these intracellular regulatory interactions, ferroptosis in CRC is further shaped by the unique intestinal microenvironment. Although the regulatory mechanisms of PCBP1/2 and ALKBH8 are not exclusive to CRC, they may be particularly relevant in this disease because ferroptosis in CRC is additionally modulated by luminal iron exposure, gut microbiota, hypoxia, and recurrent driver mutations. Luminal iron has been linked to altered NRF2-related responses and may further disturb epithelial iron homeostasis, thereby influencing the ferroptosis threshold in colonocytes [103,104]. Meanwhile, the gut microbiota can bidirectionally regulate ferroptosis sensitivity: short-chain fatty acids such as butyrate may enhance ferroptosis, whereas microbial metabolites such as indole derivatives can promote ferroptosis resistance through FSP1/CoQ10-related signaling [105,106]. In parallel, hypoxia/HIF signaling and common CRC driver alterations, including APC, KRAS, BRAF, and TP53 mutations, are also likely to intersect with iron metabolism, antioxidant defense, and lipid metabolism, thereby modifying ferroptosis vulnerability in a context-dependent manner [106,107,108]. Collectively, these factors indicate that the iron–selenium framework in CRC should be interpreted in conjunction with tumor ecological features and mutational heterogeneity.

Overall, the interrelated iron and selenium metabolic pathways help explain ferroptosis behavior in CRC. This framework has important implications for patient stratification, pharmacodynamic monitoring, and combination therapy [21,41,109,110]. However, the clinical development of specific ferroptosis inducers remains constrained by pharmacokinetic limitations and toxicity [55,85]. Implementation of pharmacodynamic monitoring is further hindered by the lack of CRC-specific ferroptosis biomarkers, the strong context dependence of ferroptosis within the tumor microenvironment, and the absence of standardized clinical methods for assessing catalytic Fe(II) activity [16,50,79]. Nevertheless, available studies already support the therapeutic potential of combined iron- and selenium-targeted strategies. For example, ACE simultaneously reduces PCBP1/2 abundance, increases Fe(II) levels, inhibits GPX4 activity, and promotes GPX4 ubiquitination and degradation, thereby disrupting antioxidant defense and outperforming classical ferroptosis inducers such as erastin and RSL3 in CRC models; in animal studies, it also shows greater efficacy than capecitabine or TAS-102 [41]. High-dose selenium-based interventions can induce ferroptosis in CRC cells by targeting the NRF2/GPX4 pathway [86], and strategies that jointly target selenium metabolism and lipid oxidation may further overcome ferroptosis resistance [21,110]. Future work should further evaluate whether multitarget strategies involving iron handling and selenium-related antioxidant defense can be implemented safely and effectively in CRC, particularly in view of potential toxicity, metabolic compensation, and context-dependent therapeutic windows [21,41,109,110].

## 6. Clinical Treatment Strategies

Ferroptosis inducers (FINs) can be broadly classified into four categories according to their mechanisms of action. The first category targets system xc-, including erastin, sulfasalazine, and sorafenib; the second directly inhibits GPX4, with RSL3 and ML162 as representative compounds; the third simultaneously depletes GPX4 and coenzyme Q10, with FIN56 as a prototypical example; and the fourth promotes ferroptosis by inducing intracellular iron overload or lipid peroxidation (Table 4) [111,112]. In CRC research, several new FINs have shown promising preclinical activity. Dual-target compounds such as ACE are representative examples [41]. The dihydroindole derivative compound 31 promotes GPX4 degradation and aggravates ROS accumulation, showing potent antitumor activity in HCT-116 cells as well as in vivo models [113]. The pyrrole derivative compound 12 induces a typical ferroptotic phenotype in HCT-116 xenografts, characterized by decreased glutathione, nicotinamide adenine dinucleotide phosphate, and reduced nicotinamide adenine dinucleotide phosphate levels, together with increased malondialdehyde and Fe(II) levels [114]. In addition, the RNA polymerase I inhibitor CX-5461 induces ferroptosis in CRC cells by promoting NRF2 ubiquitination, indirectly downregulating SLC7A11 and GPX4, and thereby collapsing antioxidant defense [115]. Some natural products also show therapeutic potential. For example, hydroxytyrosol induces ferroptosis in CRC cells through the NRF2 pathway, lowers SLC7A11 and GPX4 protein levels, and simultaneously upregulates transferrin receptor 1 and the labile iron pool [116]. These preclinical observations should not be interpreted as indicating that the iron–selenium axis alone provides a sufficient explanation for ferroptosis sensitivity in CRC, which is also shaped by GPX4-independent antioxidant systems, lipid remodeling, NADPH metabolism, and context-dependent tumor microenvironmental responses.

Although these findings support the therapeutic interest of multitarget ferroptosis-based strategies, most evidence remains preclinical, and the feasibility of simultaneously modulating iron handling and selenium-dependent defense in patients will depend on resolving substantial issues related to systemic toxicity, metabolic compensation, biomarker selection, and tumor heterogeneity. Available preclinical studies suggest that selected combination regimens may enhance ferroptotic vulnerability in CRC by perturbing iron homeostasis, antioxidant defense, or both. Examples include oxaliplatin plus erastin and salinomycin plus 5-fluorouracil, while butyrate-based sensitization approaches and nanoplatform-based systems provide additional proof of concept in specific experimental settings [14,41,117,118]. However, these findings remain insufficient to define a clinically applicable combinatorial framework, and their translational relevance will require more rigorous validation across molecular subtypes and tumor microenvironmental contexts.

Beyond direct tumor-cell killing, ferroptosis may also interact with antitumor immunity. In some settings, lipid peroxidation products and damage-associated molecular patterns released during ferroptosis can stimulate immune responses and synergize with immune checkpoint inhibitors. However, these immunological effects are highly context-dependent and still require validation in immunocompetent models and clinical samples. This issue is particularly prominent in proficient mismatch repair/microsatellite stable CRC, where response rates to immunotherapy remain low [11,119]. The gut microbiota further participates in this regulation: short-chain fatty acids can enhance ferroptosis sensitivity, whereas indole-3-acetic acid produced by anaerobic streptococci mediates ferroptosis resistance through the aryl hydrocarbon receptor-ALDH1A3-FSP1/coenzyme Q signaling axis [119]. In addition, a self-amplifying ferroptosis nanocomplex (Cu2O@Au) induces immunogenic cell death, promotes dendritic-cell maturation and T-cell infiltration, and enhances the efficacy of anti-programmed death ligand 1 therapy in CRC animal models [120]. FRG signatures are also closely related to expression of immune checkpoint genes; for example, in high-risk patients, programmed death ligand 1 and cytotoxic T-lymphocyte-associated protein 4 expression are increased, providing a theoretical basis for biomarker-guided checkpoint-based combination therapy [121]. Taken together, strategies that combine immunonanomedicine with immune checkpoint blockade may have substantial translational value and may help remodel the tumor microenvironment and reverse resistance in proficient mismatch repair/microsatellite stable CRC [119].

FRG signatures are being explored as candidate tools for prognosis assessment and treatment stratification in CRC. However, their clinical validity, ferroptosis specificity, and prospective utility remain to be established. A 10-gene FRG signature composed of TFAP2C, SLC39A8, NOS2, HAMP, GDF15, FDFT1, CDKN2A, ALOX12, AKR1C1, and ATP6V1G2 has been validated by receiver operating characteristic curve analysis and Kaplan–Meier survival analysis, shows good predictive performance, and is significantly associated with immune-cell infiltration, drug sensitivity, and somatic mutation status, including TP53 mutations [43]. A three-gene model based on GPX3, CDKN2A, and SLC7A11 also has prognostic value [122]. By integrating single-cell sequencing with bulk transcriptomic data, researchers have further identified ferroptosis dependence-related genes with good predictive ability; within this framework, high-risk CRC patients show higher levels of immune infiltration and worse clinical outcomes [123]. In stage II/III CRC, a 23-gene ferroptosis-related signature derived through machine-learning approaches can identify high-risk patients more accurately than conventional clinicopathological indicators and molecular features [124]. In addition, genetic polymorphisms in ferroptosis-regulatory genes may also have clinical significance: for example, the rs7217186 variant in ALOX15 is associated with treatment response to bevacizumab in patients with RAS-mutant metastatic CRC, suggesting that this single-nucleotide polymorphism may serve as a prognostic biomarker [22].

Although substantial progress has been made in ferroptosis research, ferroptosis-based therapeutic strategies still face multiple obstacles to clinical translation. At present, clinical development of specific ferroptosis inducers remains slow, largely because currently available agents often show limited efficacy together with considerable toxicity. Accordingly, development of new compounds with high selectivity, favorable bioavailability, and precise tumor-targeting capacity remains a central task in the field [111]. This challenge is particularly relevant for strategies that aim to simultaneously perturb iron handling and selenium-dependent antioxidant defense. In addition, the clinical value of currently approved ferroptosis-related drugs and proposed ferroptosis-specific biomarkers has not been fully established [23]. More broadly, several fundamental challenges remain: the lack of reliable biomarkers for dynamic monitoring of therapeutic response, incomplete understanding of ferroptosis regulation within the tumor microenvironment, and the possibility that ferroptosis-related damage may trigger inflammatory or immunosuppressive responses that ultimately promote tumor progression [125,126]. Tumor cells can also evade ferroptotic killing through a variety of adaptive mechanisms, including activation of the xCT-CD44v axis, upregulation of GPX4 and FSP1, induction of NRF2-dependent antioxidant programs, and mitochondrial remodeling. These changes markedly weaken therapeutic efficacy [11]. Precise control of the therapeutic window is therefore essential. On the one hand, treatment should avoid collateral injury to ferroptosis-sensitive immune cells; on the other, it must address loss of efficacy caused by antioxidant compensation within tumor cells [119,126]. Finally, the field also faces technical bottlenecks, particularly the low clinical accessibility of catalytic Fe(II) detection, which still relies largely on frozen tissues or live-cell imaging and is difficult to implement in routine clinical practice [11]. Developing clinically applicable biomarkers for patients with CRC should therefore be a major priority for future research [23].

## 7. Conclusions and Future Perspectives

This review proposes a focused iron- and selenium-centered framework for interpreting ferroptosis sensitivity in CRC, rather than a complete unified model of ferroptosis regulation. Iron- and selenium-related processes may exert opposing influences on ferroptosis and intersect functionally under selected conditions, but they should not be interpreted as a fully unified regulatory circuit. As cytoplasmic iron chaperones, PCBP1/2 not only maintain intracellular iron homeostasis and limit iron toxicity but, under specific stress conditions, can also cooperate with NCOA4-mediated ferritinophagy to release stored iron and thereby intensify ferroptotic pressure [50,55]. The NCOA4-FTH1 axis regulates ferroptosis in CRC cells through dual mechanisms: it mobilizes ferritin-bound iron to initiate ferroptosis and also interfaces with broader autophagic programs [1,42]. On the selenium side, ALKBH8 regulates selenocysteine insertion through transfer RNA modification and thereby determines the anti-lipid-peroxidation capacity of the selenoprotein network, including GPX4, TXNRD1, and SELENOI [16,18]. Ultimately, these iron- and selenium-related pathways converge on lipid peroxidation, forming a dynamic metabolic balance. NRF2 may influence ferroptosis sensitivity in CRC by intersecting with antioxidant, iron-metabolic, and lipid-metabolic programs, although its precise role remains highly context-dependent [15,97].

Despite this progress, several important questions remain unresolved. First, the temporal mechanism underlying the switch of PCBP1/2 from protective iron chaperones to ferritinophagy-promoting regulators remains unclear. In parallel, the functional differences between PCBP1 and PCBP2 across CRC subtypes, and the molecular basis of those differences, require further investigation [127,128]. Second, beyond GPX4, individual selenoproteins such as SELENOI, TXNRD1, and SELENOP appear to contribute to ferroptosis resistance in CRC cells, but their precise roles remain insufficiently defined and require more detailed mechanistic study [21,86]. From a broader clinical perspective, ferroptosis-based therapies and related biomarkers have not yet been validated in clinical trials, in part because of limited efficacy and toxicity concerns [23,129]. Moreover, the role of ferroptosis within the CRC microenvironment is highly context-dependent, and ferroptosis-related cell damage may provoke inflammatory or immunosuppressive responses that could in turn promote tumor progression and metastasis [130,131].

Even so, several emerging technologies are beginning to address these limitations. Nanotechnology-based delivery systems may improve targeted delivery of ferroptosis-modulating agents, enabling combination of chemotherapy, ferroptosis induction, and immunomodulation while reducing toxicity to normal tissues [125,132]. ATTEC-based approaches represent another highly promising direction because they enable selective ferritin degradation and iron release, thereby inducing ferroptosis with greater specificity in CRC cells [125]. In addition, proteolysis-targeting chimera strategies may selectively degrade anti-ferroptotic proteins such as GPX4, further expanding therapeutic options for ferroptosis-related disease in CRC [133]. Low-temperature plasma may also selectively induce ferroptosis in iron-rich CRC cells through generation of reactive oxygen and nitrogen species while causing limited damage to normal tissues [11,134]. Finally, combining ferroptosis-oriented nanomedicine with immune checkpoint inhibitors may be particularly valuable in proficient mismatch repair/microsatellite stable CRC [119,135].

At present, multitarget ferroptosis-oriented strategies appear conceptually promising in CRC, but their translational value remains to be established through more rigorous evaluation of efficacy, selectivity, and toxicity. Dual-target agents such as ACE strongly support this principle, as their antitumor effects are clearly superior to those of single-target approaches and therefore provide compelling evidence for multitarget ferroptosis induction [41]. Simultaneous modulation of selected selenoprotein-related pathways may merit further investigation, but its therapeutic feasibility remains uncertain because of potential toxicity, redox imbalance in normal tissues, and compensatory antioxidant responses. Compounds such as RSL3 and ML162, together with broader thioredoxin reductase inhibitors, illustrate that disruption of selenium-dependent defense systems is feasible [85,88]. In addition, FINs can be combined with conventional chemotherapeutic agents such as oxaliplatin, 5-FU, and capecitabine. By leveraging the synergy between chemotherapy-induced oxidative stress and ferroptosis-mediated collapse of antioxidant defense, such combinations may enhance therapeutic efficacy [117,120]. The connection between ferroptosis and the intestinal microbiota further suggests that precise microbiota modulation or dietary interventions that alter butyrate or selenium availability may increase tumor-cell sensitivity to ferroptosis [118,119]. Moreover, combining FINs with radiotherapy or hyperthermia may further enhance therapeutic efficacy, because both treatment modalities can increase intracellular oxidative stress, although their value in CRC still requires further validation [131].

In conclusion, ferroptosis has emerged as an important new direction in CRC research and therapy. Within the ferroptosis regulatory network, the PCBP1/2-NCOA4 axis and the ALKBH8-centered selenoprotein network are best viewed as biologically distinct regulatory layers that may intersect functionally at the level of ferroptosis output. Together, they provide a focused conceptual framework for considering how trace-element-related processes may influence ferroptosis in CRC, while remaining only one part of a broader ferroptosis-regulatory landscape. Although substantial challenges still hinder clinical translation [23,131,136], continued work should determine how this framework interfaces with other major ferroptosis-control systems, including GPX4-independent antioxidant pathways, lipid remodeling, metabolic compensation, and the tumor microenvironment, before its translational value can be fully defined.

Ultimately, only by integrating molecular subtype, driver alterations, hypoxic niche features, microbiota composition, and iron/selenium metabolic status can the ferroptosis vulnerability landscape of CRC be more precisely defined. This multidimensional perspective may facilitate the translation of the iron–selenium framework from a conceptual model into a clinically actionable strategy for precision therapy in CRC.

## Figures and Tables

**Table 1:** Major components of the canonical ferroptosis regulatory axis and their functional roles.

Regulator	Mechanism	Role in Ferroptosis	References
SLC7A11	Cystine undergoes reverse exchange with glutamate via SLC7A11 to maintain intracellular GSH levels and exert antioxidant effects.	Prevents the accumulation of lipid peroxides by maintaining GSH homeostasis, thereby inhibiting the occurrence of ferroptosis.	[8,9,11,14]
GPX4	Catalyzes the reduction of lipid peroxides to block the lipid peroxidation cascade induced by the Fenton reaction.	Directly inhibits the process of lipid peroxidation and prevents the initiation and occurrence of ferroptosis.	[8,11]
FSP1	Regulates ROS levels and combines with CoQ10 to synergistically inhibit lipid peroxidation, thereby alleviating ferroptosis-related effects.	Reduces ROS production and protects cells through antioxidant effects to achieve the inhibition of ferroptosis.	[8,9,10,11,12]
FRGs	Maintains cellular iron homeostasis by regulating transmembranous iron transport and ferritin synthesis.	Highly expressed FRGs promote ferroptosis, while lowly expressed FRGs reduce cellular sensitivity to ferroptosis.	[11]

Note: SLC7A11, solute carrier family 7 member 11; GSH, glutathione; GPX4, glutathione peroxidase 4; FSP1, ferroptosis suppressor protein 1; CoQ10, coenzyme Q10; ROS, reactive oxygen species; FRGs, ferroptosis-related genes.

**Table 2:** The PCBP1/2–NCOA4 axis in ferroptosis regulation.

Regulator	Mechanism	Role in Ferroptosis	References
PCBP1	Acts primarily as a cytosolic iron chaperone that delivers iron to ferritin and other target proteins; possible links to ferritinophagy-related processes have been proposed in selected stress settings.	Current evidence supports a predominantly protective iron-buffering role, although context-dependent pro-ferroptotic effects have also been proposed under specific stress conditions.	[16,18,19,21,23,41,50,55]
PCBP2	Supports intracellular iron trafficking and iron homeostasis; its contribution to ferroptosis-related iron redistribution appears to be context-dependent.	Likely context-dependent; may support iron homeostasis under basal conditions but contribute to ferroptosis-related iron redistribution in selected stress settings.	[19,21,23,45,46,47,48,49]
NCOA4	Interacts with PCBP1 to promote ferritin degradation and iron release.	Promotes ferroptosis by expanding the labile iron pool and Fe(II) accumulation.	[16,19,21,41,50,54,55,56]

Note: PCBP1/2, poly(rC)-binding proteins 1 and 2; NCOA4, nuclear receptor coactivator 4.

**Table 3:** Selected ALKBH8-associated and selenium-linked regulators relevant to ferroptosis.

Regulator	Mechanism	Role in Ferroptosis	References
ALKBH8	Modifies tRNA and influences selenocysteine insertion efficiency, thereby controlling selenoprotein production.	Promotes the synthesis of selenoproteins such as GPX4 and TXNRD1, enhances antioxidant capacity, and suppresses ferroptosis.	[16,17,18,21,41,50,55,85]
PRDX6	Acts primarily in redox regulation and phospholipid metabolism.	Any direct role in selenium handling remains hypothetical.	[16,17,18,21,23,50,55,85]
GPX4	Reduces lipid peroxides to maintain cellular redox homeostasis and prevent ferroptosis.	Directly blocks lipid peroxide accumulation and the execution of ferroptosis.	[8,11,16,18,21,41,50,85]

Note: ALKBH8, alkB homolog 8; PRDX6, peroxiredoxin 6; GPX4, glutathione peroxidase 4; TXNRD1, thioredoxin reductase 1.

**Table 4:** Classes of ferroptosis inducers and their mechanisms relevant to CRC therapy.

Regulator	Representative Agents	Mechanism	Role in Ferroptosis	References
Class I inhibitors	Erastin; sorafenib	Inhibit system xc^−^-mediated cystine uptake, deplete intracellular glutathione, and weaken GPX4-dependent peroxide detoxification.	Represents a candidate strategy for impairing cystine-dependent antioxidant defense, although supporting evidence in CRC remains largely preclinical.	[8,11,21,41,85,101]
Class II inhibitors	RSL3; ML162	Directly inhibit GPX4 activity, thereby impairing lipid peroxide detoxification and promoting ferroptotic damage.	Provides a direct pharmacologic approach to GPX4-dependent ferroptosis induction, but its relevance to chemoresistant CRC remains to be validated in broader preclinical and translational settings.	[8,11,16,21,41,85]
Class III inhibitors	FIN56	Promote ferroptosis by simultaneously weakening GPX4-associated antioxidant protection and coenzyme Q10-related defense capacity.	May have relevance in chemoresistant settings by simultaneously weakening multiple antioxidant defenses, although supporting evidence in CRC remains largely preclinical.	[8,11,16,21,41,85]
Class IV inhibitors	CoQ10; salinomycin	Promote ferroptosis by increasing intracellular iron availability and/or enhancing lipid peroxidation-associated oxidative stress.	May be particularly relevant in tumors with disturbed iron homeostasis, but context dependence, systemic toxicity, and tumor heterogeneity should be taken into account.	[1,11,16,19,41,50]

Note: GPX4, glutathione peroxidase 4; CoQ10, coenzyme Q10.

## Data Availability

No new data were created or analyzed in this study. Data sharing is not applicable to this article.

## Abbreviations

The following abbreviations are used in this manuscript:

CRC	colorectal cancer
GPX4	glutathione peroxidase 4
SLC7A11	solute carrier family 7 member 11
PCBP1/2	poly(rC)-binding proteins 1 and 2
NCOA4	nuclear receptor coactivator 4
ALKBH8	AlkB homolog 8
ROS	reactive oxygen species
GSH	glutathione
xCT	cystine/glutamate antiporter
CD44v	CD44 variant subtype
FSP1	ferroptosis suppressor protein 1
NRF2	nuclear factor erythroid 2-related factor 2
LIP	labile iron pool
PRDX6	peroxiredoxin 6
RNA	ribonucleic acid
SELENOI	selenoprotein I
ACE	acevaltrate
FRG	ferroptosis-related gene
DNA	deoxyribonucleic acid
ALOX15	arachidonate 15-lipoxygenase
BECN1	beclin 1
ATM	ataxia-telangiectasia mutated kinase
FTH1	ferritin heavy chain 1
FTL	ferritin light chain
HERC2	HECT and RLD domain containing E3 ubiquitin protein ligase 2
VAMP8	vesicle-associated membrane protein 8
ATG5	autophagy related 5
ATG7	autophagy related 7
UGA	UGA stop codon
EEFSEC	eukaryotic elongation factor, selenocysteine-tRNA-specific
TXNRD1	thioredoxin reductase 1
SELENOP	selenoprotein P
LOOH	lipid hydroperoxides
BACH1	BTB and CNC homology 1
5-FU	5-fluorouracil
BRAF	B-Raf proto-oncogene, serine/threonine kinase
TFAP2C	transcription factor AP-2 gamma
SLC39A8	solute carrier family 39 member 8
NOS2	nitric oxide synthase 2
HAMP	hepcidin antimicrobial peptide
GDF15	growth differentiation factor 15
CDKN2A	cyclin-dependent kinase inhibitor 2A
ALOX12	arachidonate 12-lipoxygenase
TP53	tumor protein p53
GPX3	glutathione peroxidase 3
